# Politically Motivated Internet Addiction: Relationships among Online Information Exposure, Internet Addiction, FOMO, Psychological Well-being, and Radicalism in Massive Political Turbulence

**DOI:** 10.3390/ijerph17020633

**Published:** 2020-01-18

**Authors:** Gary Tang, Eva P. W. Hung, Ho-Kong Christopher Au-Yeung, Samson Yuen

**Affiliations:** 1 Department of Social Science, The Hang Seng University of Hong Kong, Hong Kong 999077, China; garytang@hsu.edu.hk (G.T.); christopherauyeung@hsu.edu.hk (H.-K.C.A.-Y.); 2 Department of Political Science, Lingnan University, Hong Kong 999077, China; samsonyuen@Ln.edu.hk

**Keywords:** Internet addiction, depression, radicalization, social movement, Hong Kong

## Abstract

This research examines the mediating role of the tendency for Internet addiction, fear of missing out (FOMO), and psychological well-being in the relationship between online exposure to movement-related information and support for radical actions. A questionnaire survey that targets tertiary students was conducted during the Anti-Extradition Law Amendment Bill (Anti-ELAB) Movement (N = 290). The findings reveal the mediating effect of Internet addiction and depression as the main relationship. These findings enrich the literature of political communication by addressing the political impact of Internet use beyond digital architecture. From the perspective of psychology, this research echoes the literature that concerns depression symptoms driven by a protest environment. Radical political attitudes driven by depression during protests should also be concerned based on the findings of this survey.

## 1. Introduction

The political impact of the Internet is a major topic in political communication as it may affect voting behavior, political discussion, and political participation, etc. Most research so far has focused on the role of alternative information and the impact of network structures on social media, which is believed to affect actors’ decision-making based on the information and opinion to which they are exposed [1,2,3]. However, besides the impact of exposure to information and opinion, the political impact of the Internet can be studied from a more everyday life perspective.

The political implications of the Internet can be more revealing during times of social and political turbulence. In a massive protest which is emotionally provoking and full of unexpected social and political dynamics, some people are prompted to keep being exposed to information related to the protest. This tendency can be addictive among them. The impact of Internet addiction on psychological well-being is a widely studied topic. In the ongoing Anti-Extradition Law Amendment Bill (Anti-ELAB) Movement in Hong Kong, the society is polarized regarding some radical and militant protests, and thousands of protesters were arrested. Perceived social isolation and depression, as attributes of psychological well-being, are worth investigating in this protest situation. Some people might perceive themselves to be isolated minorities during the addictive exposure to the online public opinion. Others might feel depressed from seeing some protesters sacrificed for the movement in terms of being arrested and beaten up by the police, etc. The relationship between depression and support for radical actions is a new area of research that is under-explored. The Anti-ELAB Movement is characterized for being sentimentally eventful and radicalized with persistent support from parts of the public [4]. This movement provided an optimal background for data collection and analysis for this exploratory research. Therefore, with the data collected from students at the tertiary level during a relatively mild period in the movement, this research attempts to explore the relationship among the use of Internet for movement-related information, Internet addiction, psychological well-being, and support for radical actions.

## 2. Theoretical Framework

The political impact of the Internet on social movements is an important theme in political communication, and there has been a vast amount of literature on the relationship between the Internet and social mobilization. The topic is especially important when many massive protests were leaderless, uncertain, and improvising in the age of social media, such as the Arab Spring uprisings and the global Occupy movement. Explanations related to the Internet and social media were provided. The Internet is an open platform for developing a vibrant landscape of alternative media, where alternative views and information on social movements that are commonly ignored by conventional news media can be widely circulated [2,5]. Frequent viewers of alternative news were also found to be more knowledgeable in protest-related information [6]. Therefore, the Internet can bring a positive effect on social mobilization and cultivate a stronger acceptance of radical actions among frequent viewers of alternative opinions.

The second stream focuses on the structure of social networking on social media. The architecture of social media is favorable to exposing people to opinions and information of a similar rather than opposing view. It allows minorities with alternative views to overestimate their influence, discourages people with opposing views to be exposed to each other, and encourages an echo-chamber effect [3,7,8]. The online networking platform also helps strengthen their identity and group efficacy for mobilization when they can easily connect with each other [9,10,11]. Moreover, social media facilitates the mobilization of “connective action” that is crowd-enabled, leaderless, and improvised [12,13,14].

Following the trend of the “affective turn” [15], affect and emotions are increasingly considered when discussing the role of the Internet in political communication [16]. The nature of networking is still key to the analysis. Papacharissi [17] gave a comprehensive account of how affect is circulated on social media, and how an affective-networked public is built. Studying university students in urban China, Gan, Lee, and Li [18] found that circulation of negative emotions through “public affair communication via social media” strengthens the impact of social media on political participation. Kim and Kim [19] further found that negative emotion is strengthened when users are exposed to dissimilar views together with uncivil comments.

The emphasis on the nature of the Internet and social media in political communication is due to the background of technological determinism of communication studies [20]. The supposedly unlimited amount of information on the Internet drives scholars to compare the impact of online news media to the conventional news media’s ability to set the agenda [21]. The “selective exposure” thesis proposed by Bennett and Iyengar [22] also defines the way of studying the impact of the Internet on public opinion and political attitudes. Against this background, this research attempts to examine the relationship between Internet use and political attitudes by filling the following gaps in the literature. First, instead of considering the infrastructural nature of the Internet and social media to be the analytical premise, this research adopted a user-centered approach by focusing on the experience and psychological impact of obtaining news information online. Second, variables about psychological well-being are often considered as the ultimate dependent variables in a psychological study. This is due to the practical purpose of clinical psychology, which inclines to identify individuals that are more vulnerable to problems related to psychological well-being. However, this research attempts to examine the association between psychological well-being and political attitude, and the possible mediating role of the former. Third, although there is a large volume of literature related to the impact of the Internet on political attitudes and political participation, its impact on radicalization is rarely discussed. Fourth, there are only limited studies on the psychological and attitudinal impacts of the Internet conducted during a protest. To address the above, this research includes the tendency of Internet addiction, fear of missing out (FOMO), and psychological well-being to examine the relationship between Internet use and support for radical actions.

Internet addiction is highlighted because it is closely relevant to a social movement context, especially prolonged large-scale political incidents. Many remarkable massive social protests were emotionally eventful in the sense that they are composed of sentimental moments. Emotionally driven actions during protests can affect the overall dynamics between protesters and the government. Examples can be found in major massive protests in the Arab Spring [23]. In Hong Kong, the Umbrella Movement in 2014 was also found to be motivated by unusual grievances and it had a continuous psychological impact on the population across the movement [24,25]. During a massive protest, the context itself can motivate people to have stronger tendency to be exposed to movement-related information. In its extreme form, Internet addiction can be expected within the context of a movement.

Internet addiction refers to an extreme degree of obsession to the use of the Internet in the sense that users’ offline social life is interrupted because of indulgence in the online arena. It is an important topic in clinical psychology as Internet addiction can bring a range of problems in people’s psychological well-being. Uses of the Internet, including online gaming and social networking addiction, have been widely studied [26]. On the other hand, FOMO can be an amplified practice of Internet addiction. FOMO refers to “pervasive apprehension that other might be having rewarding experiences from which one is absent” [27]. It implies an anxiety of being out-grouped among one’s peers in terms of circulation of information that is considered to be valuable for in-group bonding. Being an emotional symptom, FOMO clearly does not just take place online; however, in the online realm, FOMO can intensify problematic use of social media that affects the well-being of both the users and their peers [28].

The impact of Internet addiction and FOMO on psychological well-being has been widely examined. The research of Wu, Cheung, Ku, and Hung [29] illustrated the positive relationship between the use of social networking sites and its addictive tendency, and it addressed the consequent psychological risk. Studying the addictive use of smartphones, Peper and Harvey [30] found that addictive use of smartphones strongly leads to a range of mental health symptoms, including depression, anxiety, loneliness, etc. People with heavier social media use were found to have stronger perceived social isolation and depression, respectively [31,32]. Similar observations were also found in FOMO. The experiment of Hunt, Marx, Lipson, and Young [33] revealed that FOMO and its associated depression can be reduced by limiting the use of social media. Robinson, Bonnette, Howard, Ceballos, Dailey, Lu, and Grimes [34] explained the relationship between Internet addiction and depression in more detail based on a comprehensive survey. They found that Major Depressive Disorder was more likely to be found among people who had a stronger tendency to compare themselves with other people on social media, who were more eager to maintain a positive image on social media, and had a larger perceived discrepancy between their online and offline identity.

To explore the relationship between Internet use and the support for radical actions by a user-centered approach, the above psychological variables are considered. Not only have their relationships been supported, but they are also worth discussing in the context of social movements. First, as mentioned, a massive protest usually includes sentimental moments that induce people to keep themselves updated about the protest. Second, a massive protest is an important event in the public agenda as well as a popular topic for public discussion. People can feel the need to keep updated about the event in order to feel involved in the group alongside their peers. Referring to the observation of Robinson et al. [34], Internet addiction in the context of a movement can lead to depression due to comparison between users and their peers who are highly involved in the movement and adjusting one’s persona on social media to fit the movement atmosphere. Third, a movement context itself can be emotionally stimulating. A massive protest is an amalgam of various emotions including grievances, anxiety, hope, desperation, etc. [35]. The impact of addictive exposure to movement-related information can be strengthened in this context. Fourth, given that emotions have a significant place in social mobilization [36], the psychological well-being driven by addictive exposure potentially has a similar role in enhancing support for radical actions.

Based on the above discussion, the following research questions and hypotheses are proposed to substantiate the analysis of the relationship between Internet use and support for radical actions.

The first hypothesis aims at testing the direct relationship between Internet use and support for radical actions. A positive relationship is partly supported by the research of Lee [37], though his findings focused on the exposure to online alternative media that facilitates the spread of radical views. Therefore, focusing on exposure to movement-related information online in a general sense, *H1* aims at opening the discussion by testing their direct relationship:

*H1:*  Online information exposure and support for radical actions are positively related.
*H2:*  and *H3* try to echo the relationship between online information exposure, Internet addiction, and psychological well-being that has been discussed in psychology. FOMO is also included as it is associated with Internet addiction [38] and it fits the movement context that is full of uncertainties and unexpected incidents. Psychological well-being includes depression and perceived social isolation as they are commonly observed as possible consequences of Internet addiction [29,31]. In the context of a movement, depression was found to be an emotional symptom during and after a protest [39,40], but its role is rarely discussed in social mobilization. In addition, perceived social isolation is not covered in the discussion about mobilization and yet, addictive Internet use can lead to a perception of whether some people are part of the majority or minority in a highly polarized atmosphere during a massive protest. The operationalization of the measurements was adjusted due to the context of massive protest in Hong Kong. This will be explained in the next section.
*H2a:*  Online information exposure relates positively to tendencies of Internet addiction.
*H2b:*  Online information exposure relates positively to FOMO.
*H3a:*  Internet addiction relates positively to depression.
*H3b:*  Internet addiction relates positively to perceived social isolation.
*H3c:*  FOMO relates positively to depression.
*H3d:*  FOMO relates positively to perceived social isolation.

The respective impact of depression and perceived social isolation on support for radical actions can be made sense of in the context of a movement. Feelings of depression are more ambiguous than anger, fear, and hope which are more frequently discussed in social mobilization. Depression is a mix of negative feelings including sadness, self-disappointment, inefficacy, guilt, etc. Regarding the relationship between depression and radicalism, there was research that investigated how depression as a mental illness related to extreme behaviour and terrorist attacks [41]. However, the relationship between depression and support for radical actions as a general political attitude is rarely discussed. The relationship between the two can be both positive and negative conceptually. Depression can be positively related to support for radical actions as people with stronger depression may seek a more radical change to overcome their depression. In contrast, depression can discourage people from supporting radical actions due to the feeling of powerlessness. Similarly, there was no clear evidence about the impact of perceived social isolation on supporting radical actions. In public opinion studies, people who perceived themselves to be the minority were found to be silent rather than vocal and radical [42]. On the other hand, some people with stronger perceived social isolation consider themselves to be the legitimate frontier in the context of a movement and tend to show stronger support for radical actions. Due to the open possibilities regarding the impact of depression and perceived social isolation, the following research questions are proposed:

*RQ1:*  RQ1: What is the relationship between depression and support for radical actions?
*RQ2:*  What is the relationship between perceived social isolation and support for radical actions?

The last hypothesis is established based on the potential findings of the above hypotheses and research questions, and the literature arguing that the relationship between Internet use and political attitude and participation is intervened by other variables rather than a direct relationship [43,44].

*H4:*  The relationship between online information exposure and support for radical actions is stronger when it is mediated by Internet addiction, FOMO, and psychological wellbeing.

## 3. Materials and Methods

### 3.1. Key Variables

*Online information exposure*. The respondents were asked about their frequency of obtaining movement-related information from a list of media outlets. They answered for each media outlet from 1 = “never” to 5 = “always”. The frequency of obtaining movement-related information through online news media, Facebook, Instagram, WhatsApp, Snapchat, Telegram, and online forums was averaged to construct the variable (*α* = 0.80, *M* = 3.73, *SD* = 0.87).

*Internet addiction*. The index of Internet addiction was referenced from the Young’s Internet Addiction Test [45]. The measurements were moderated to match the context of a movement. The respondents were asked to what extent they agree with the following statements: “I spend less time with family and friends because I spend time on surfing movement-related information online”, “I expect to see updated information about the movement online”, “I feel pissed when I am interrupted by the other from surfing movement-related information online”, “I cannot sleep at night because I spend time surfing movement-related information online”, and “my study and work are affected because I spend much time on surfing movement-related information online”. The respondents answered from 1 = “never” to 5 = “always”. The index was constructed by averaging the five statements (*α* = 0.86, *M* = 2.75, *SD* = 1.06).

*FOMO*. Measurement of FOMO was referenced from Przybylski et al.’s [27] 10-item FOMO-scale. Four of them, which were more relevant to the context of a movement, were chosen and revised to be covered in this survey. The respondents were asked how frequently they experienced the following: “I feel bad when my friends know more information about the movement than I do”, “sometimes I find that I spend too much time on knowing the latest information about the movement”, “sharing updated information about the movement (both online and offline) with friends is an important thing for me”, and “I am eager to know updated information about the movement when I am on a vacation”. The respondents answered from 1 = “never” to 5 = “always”. The index was constructed by averaging the score from the four statements (*α* = 0.74, *M* = 2.47, *SD* = 0.92).

*Perceived social isolation*. This set of measurement was referenced from Primack et al. [31] with minor moderations in translation. The respondents were asked to what extent the following statements describe the relationship between the respondent and his/her friends in the Anti-ELAB Movement: “I feel that I am ignored by them”, “I feel that they cannot understand me”, “I feel that I am too different from them”, and “I feel that my friends and I belong to different circle”. The respondents answered from 1 = “totally cannot” to 5 = “totally can”. The variable was constructed by averaging the score from the four statements (*α* = 0.89, *M* = 1.71, *SD* = 0.83).

*Depression*. Five sets of measurements from The Beck Depression Inventory [46] that fit the situation of young people and the context of the movement were used in this survey. The respondents were asked “in each set of statements, please choose a statement that can describe your feeling in this movement”. The statements can be found in Table 1. Each statement refers to different degree of depression, from 1, meaning no sign of depression, to 4, meaning serious signs of depression. The variable was constructed by averaging the score of the five sets of statements to a 1 to 4 index (*α* = 0.73, *M* = 2.06, *SD* = 0.52).

*Support for radical actions*. The respondents were given a list of radical protest actions which took place during the Anti-ELAB Movement, and they were asked to what extent can they accept the actions, respectively. They answered from 1 = “strongly unacceptable” to 5 = “strongly acceptable”. The list of actions includes “blocking roads”, “interrupting the operation of railway and airport”, “vandalizing railway stations and facilities inside the station”, “vandalizing shops owned by Chinese capital or owners who openly expressed pro-government opinion”, “arson”, “doxing police officers”, “militant attack to policemen”, and “physically attacking people with opposite views who attacked or making nuisance to other protesters (private resolution)”. The variable was constructed by averaging the degree of acceptance of all the actions (*α* = 0.96, *M* = 3.41, *SD* = 1.15). Furthermore, the respondents’ support for “ordinary peaceful protests” was also covered in the survey which is addressed in the analysis for reference (*M* = 4.74, *SD* = 0.65).

Besides demographics, two variables related to attitude and participation in the Anti-ELAB Movement were included in the analysis as control variables.

*Support for Anti-ELAB Movement*. The respondents were asked how much they support the Anti-ELAB Movement in general, and they answered from 1 = “totally not support” to 10 = “totally support” (*M* = 8.02, *SD* = 2.35).

*Participation in theAnti-ELAB Movement*. This variable is constructed based on two questions. First, 12 major protests from 9 June to 20 October were listed, and the respondents were asked to indicate the protests they attended, which gave us a continuous variable for participation in major protests from 0 to 12. Besides the major mass protests, there were numerous protests at the community level that took place in different districts since July. The respondents were asked how many of those protests they attended. They answered from 0 = “none” to 4 = “more than 10”. The variable was constructed by summing the number participating in major mass protests and community-level protests (*M* = 3.76, *SD* = 4.37).

### 3.2. Background and Data Collection

This research was conducted in Hong Kong during the fifth month of the Anti-ELAB Movement, which is still ongoing at the time of writing. The movement has been eventful in the sense that it witnessed a lot of critical moments that changed the state–society relationship and citizens’ political attitudes. It was initially mobilized to urge the government to withdraw the Extradition Bill that could seriously harm the rule of law and liberty in Hong Kong. At a later stage, due to the intensification of conflicts between the police force and citizens, the movement was also driven by public dissatisfaction with the police force [4]. Many citizens were emotionally stimulated by the brutality of the police in the protest every weekend. Research on data traffic of a major online forum “LIHKG” from June to August shows that the number of visits rocketed every Monday and Tuesday due to people revisiting the events that occurred during the previous weekend (the data from the online forum were collected and analysed by some authors of this manuscript. Relevant details are in another manuscript that is currently under review). According to a longitudinal survey carried out by the School of Public Health, The University of Hong Kong [47], the degree of depression of Hong Kong citizens during this movement reached an all-time high since the survey began in 2009. Both evidently showed that the Anti-ELAB movement is an emotionally stimulating event that affects one’s psychological well-being and political attitude. This movement can serve as an appropriate background to study the relationship between Internet use, psychological well-being, and support for radical actions.

Young protesters are the core members of the Anti-ELAB movement [4], and therefore this research solely focuses on tertiary students in Hong Kong. The period of data collection was from 4 to 8 November 2019, which was a relatively mild period in the movement. To ensure diversity of samples, data were collected from various tertiary educational institutes in Hong Kong, which included both universities as well as colleges that offer sub-degree programs. Teachers from each participating institute presented a QR code for the online questionnaire during class breaks and students took part on a voluntary basis. Most of the samples were collected in classes of the General Education curriculum for a better sample heterogeneity. Coffee coupons were offered to respondents to encourage participation.

A total of 290 samples were collected. As all questions were set to be mandatory at the digital interface, the completion rate is 100%. Over half of the samples (59.3%) were from females and most of the respondents were aged between 18 and 20 (78.6%). Eighty percent were born in Hong Kong. Regarding their socio-economic status (SES), 26.1%, 27.6%, and 37.4% claimed that they were from the lower, lower-middle, and middle classes, respectively. Most of the respondents identified themselves to be moderate democrats (40.1%) and 21.7% were localists under a broad definition.

## 4. Results

Table 2 shows the zero-ordered correlations of key variable before hypotheses testing. Both attitudinal support for, and participation in, Anti-ELAB are positively correlated with online information exposure (*r* = 0.51 and 0.40 respectively, *p* < 0.001 for both). It supports the observation that people with stronger involvement in the movement tend to be more attentive to the relevant information, albeit not being part of the main analysis. Internet addiction and FOMO are highly correlated (*r* = 0.75, *p* < 0.001), which is consistent with the literature. There is no significant correlation between depression and perceived social isolation although they are both considered to be related to psychological well-being. This result is not unexpected as depression can also be intensified by mutual reinforcement among peers. Besides perceived social isolation, all the key variables are positively related to support for radical actions (*r* = 0.47 to 0.79, *p* < 0.001 for all).

Models 1 to 3 of Table 3 show the relationships between the independent variables and support for radical actions, while Models 4 to 6 of the same table show the regression analysis for support for peaceful protests that is used as reference. As expected, attitudinal support for the movement strongly associates with support for radical actions (*β* = 0.63, *p* < 0.001 in Model 1). There is also a statistically significant but weak relationship with support for peaceful protests (*β* = 0.18, *p* < 0.01 in Model 4). Online information exposure has no significant relationship with support for radical actions and therefore *H1* is rejected. Interestingly, online information exposure relates positively to support for peaceful protests with an impressive strength (*β* = 0.38, *p* < 0.001 in Model 4). This means that online information exposure can prompt people to support social protests only in a general sense. Neither Internet addiction nor FOMO has a significant relationship with support for radical protests, and they have no significant relationship with support for peaceful protests either. Model 3 tests *RQ1* and *RQ2*. It shows that people with stronger depression tend to support radical actions more (*β* = 0.13, *p* < 0.01). However, those with stronger perceived social isolation tend to show less support for radical actions (*β* = −0.07, *p* < 0.05). The latter finding echoes the spiral of silence thesis that perceived minorities tend to stay mild instead of being vocal [42].

Table 4 shows the relationship among online information exposure, Internet addiction, FOMO, depression, and perceived social isolation by regression analysis. People with stronger attitudinal support for the movement have a higher degree of depression (*β* = 0.31, *p* < 0.001). People with more online information exposure have a stronger Internet addiction and FOMO (*β* = 0.29 for both, *p* < 0.001 for both) and hence *H2a* and *H2b*, respectively, are supported. While online information exposure has no direct impact on depression, Internet addiction relates to depression positively (*β* = 0.28, *p* < 0.001). FOMO has no impact on depression. Neither Internet addiction nor FOMO is related to perceived social isolation. Only *H3a* is supported among the whole set of *H3*.

**Figure 1:**
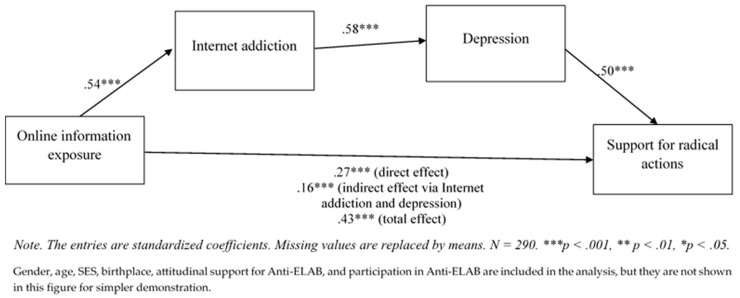
Analysis of mediating effect of Internet addition and depression on online information exposure and support for radical actions.

Table 3 and Table 4 provide a preliminarily positive sign for testing the mediating effect. The results of Sobel’s test show that Internet addiction is a significant mediator between online information exposure and depression (*z* = 3.08, *p* < 0.01), while depression is a significant mediator between Internet addiction and support for radical actions (*z* = 2.29, *p* < 0.05). Therefore, *H4* is partly supported. Figure 1 illustrates the path of mediating effect by structural equation modelling. To improve the model, FOMO and perceived social isolation were removed while all the other control variables were kept. The goodness of fit is short of being optimal (RMSEA = 0.24, CFI = 0.65, *X^2^* = 344.01, *p* < 0.05). Nevertheless, the findings in structural equation modelling can support the mediating role of Internet addiction and depression that was found to be significant. A bootstrap test with bias-corrected 95% confidence intervals on 5000 bootstrapped samples was conducted to further substantiate the result. The result shows a significant indirect effect of Internet addiction and depression on the relationship between online information exposure and support for radical action (*β* = 0.16, *p* < 0.001).

## 5. Discussion

This research explores the relationship between Internet use, addictive behaviour, psychological well-being, and radicalism. The findings open doors for further research on political communication, psychology, and social movement studies. First, this research provides a concrete explanation for the relationship between online information exposure and support for radical actions, which is still an under-explored dimension in both political communication and social movement studies. Besides the influence of content such as alternative news and similar opinions, this research shows that the psychological process of addictive Internet use to depression will lead people to support radical actions. The user-centered approach argues that a protest atmosphere itself can drive people to become addictive in obtaining movement-related information, which indirectly brings stronger acceptance of radicalization. However, it does not mean that contents and opinions circulating on the Internet are minor issues. Instead, Internet addiction can be strengthened by exposure to certain types of contents and opinion. The relationship between Internet addiction and the type of contents circulating online can be a meaningful topic for follow-up research to strengthen the understanding of how radicalization in a protest can be influenced by online communication.

Second, the significant relationship between depression and support for radical actions is the key contribution of this research. While the role of some emotions including anger, grievances, desperation, hope, anxiety, etc., in mobilization and radicalization has been widely explored, the coverage of depression is still thin. It is probably because depression is more readily associated with demobilization rather than making a positive impact. Yet, this research shows the potential to further investigate the political implication of depression. Regarding its relation to support for radical actions, a possible qualitative explanation can be drawn from our observation in the Anti-ELAB movement. That is, the feeling of depression amongst certain people was based on the perception and experience that they could not contribute much, but at the same time witnessing the sacrifice that many people made during the movement. They therefore would support the people who attempted to fight for the goals through alternative and radical means. In this sense, the positive relationship between depression and support for radical actions includes demonstration of solidarity and sense of compensation. This can also explain why attitudinal support for the movement relates to depression positively, yet the latter has no relationship with frequency of participation. In this sense, the study of depression within a social movement is beyond the interests of public health and clinical psychology and deserves further investigation in social movement studies.

Third, this research opens a new dimension to study Internet addiction. Given that the online realm represents a “virtual realm” of society that “authentic” social life could be interrupted by Internet addiction, studies related to psychological risk from Internet addiction tend to focus on users’ detachment from social life and its impact on personal and public health. This research reveals that the risk of depression symptoms can also come from online addiction due to an intensive alert to a society in turbulence. This finding represents an alternative paradigm to study Internet addiction. More importantly, in the age of social media and leaderless “connective action”, politically motivated Internet addiction will be more prevailing rather than declining. Figuring out those who are vulnerable to depression and who need professional advice during a protest is a practical concern for clinical psychologists. While the relationship between depression and extremism and terrorist actions is still debatable [41], this research found that depression and support for radical actions as political attitude are associated. Follow-up research is necessary to further examine those who are more likely to conduct deviant and radical activities due to feelings of depression.

The limitations of this research must be addressed. First, being a cross-sectional survey conducted during a protest, respondents’ feelings and attitudes can inevitably only be recorded at a particular time. During a prolonged and eventful movement like the Anti-ELAB movement, people’s emotions fluctuate as unexpected incidents unfold. This research was conducted during a relatively calm period before a major incident broke out the following week. This explains why the respondents’ degree of depression was relatively less serious. In an ideal situation where data can be collected shortly after a significant incident, a comparison between the models in a normal situation during a protest and shortly after an incident would be possible.

Second, the overall sample size is somewhat dissatisfactory. This is due to a series of major incidents that took place during the second week of November, which led to the cancellation of all remaining classes of the teaching semester in most tertiary institutes. This clearly has a significant impact on data collection. The weakness is that descriptive findings are not very referential because of the margin of error. However, this does not weaken the value of the multivariate analysis as it still brings a lot of insights for follow-up research. Key variables can be replicated in surveys conducted during the later stage of the movement and even in normal situations. Moreover, although young people with a tertiary level of education made up the main body of the anti-ELAB movement [4], the psychological well-being and political attitudes of secondary school students should also be investigated, as they face more layers of pressure from both school and family.

Third, being a cross-sectional survey means that the inter-relationships of some variables are hypothesized to be casually related instead of empirically tested by experiment. Having said that, the path from online information exposure to support for radical actions as indicated in the study forms the foundation of the follow-up experiment. What kind of content can stimulate stronger Internet addiction? To what extent depression is a consequence of Internet addiction rather than a motivation of Internet addiction? These are all interesting questions to be investigated by carrying out experiments. In this case, this paper opens up a new path to study online addiction and depression in the context of a movement.

Fourth, the measurements of Internet addiction, FOMO, perceived social isolation, and depression were modified based on the existing scales of which their reliability is promising [27,31,45,46]. The modification enables the analysis to be more focusing on people’s everyday life and mental condition under a context of movement. However, their validity can be uncertain when the context of movement is emphasized in the operationalization. Respondents’ answers can go extreme if there is an unexpected incident taken place during the data collection period. Researchers need to pay attention to this limitation when follow-up research on similar topics is conducted.

## 6. Conclusions

This research aims to examine the relationship between online exposure to movement-related information and support for radical actions during a social movement. In this research, their relationship was found to be stronger when they are mediated by Internet addiction and depression. People with more online exposure to movement-related information are more likely to have Internet addiction. The latter is positively related to depression. Then, stronger depression prompts stronger support for radical actions. This finding has profound implications for political communication, social movement studies, and political psychology, as it is an exploratory attempt to adopt psychological well-being to be an intervening variable in order to explain the relationship between exposure to online information and radicalism. Besides this key finding, several findings outside the major argument should be noted. First, participation in the Anti-ELAB movement relates positively to support for radical actions, but it has no relationship with support for peaceful protests. In contrast, online exposure to movement-related information relates positively to support for peaceful protests, but it has no relationship with support for radical actions. Third, a strongly perceived level of social isolation discourages people from supporting radical actions.

## Figures and Tables

**Table 1:** Operationalization of depression.

	1 Point	2 Points	3 Points	4 Points
Answer set 1	I do not feel sad	I feel sad	I feel sad all the time	I feel so sad that I cannot stand it
Answer set 2	I am not particularly discouraged about the future	I feel discouraged about the future	I feel I have nothing to look forward to	I desperately feel the future is hopeless
Answer set 3	I get as much satisfaction out of things as I used to	I don’t enjoy things the way I used to	I don’t get real satisfaction out of anything anymore	I am dissatisfied or bored with everything
Answer set 4	I don’t feel particularly guilty	I feel guilty a good part of the time	I feel quite guilty most of the time	I feel guilty all of the time
Answer set 5	I don’t feel disappointed in myself	I am disappointed in myself	I am disgusted with myself	I hate myself

**Table 2:** Correlations of key variables.

	(1)	(2)	(3)	(4)	(5)	(6)	(7)
1. Attitudinal support for Anti-ELAB	--	--	--	--	--	--	--
2. Participation in Anti-ELAB	0.55 ***	--	--	--	--	--	--
3. Online information exposure	0.51 ***	0.40 ***	--	--	--	--	--
4. Internet addiction	0.59 ***	0.54 ***	0.54 ***	--	--	--	--
5. FOMO	0.52 ***	0.49 ***	0.50 ***	0.75 ***	--	--	--
6. Depression	0.57 ***	0.46 ***	0.41 ***	0.58 ***	0.50 ***	--	--
7. Perceived social isolation	−0.10	0.068	0.021	0.13 *	0.15 *	0.01	--
8. Support for radical actions	0.79 ***	0.63 ***	0.47 ***	0.60 ***	0.51 ***	0.60 ***	−0.09

Note. Missing values are replaced by means. N = 290. *** *p* < 0.001, ** *p* < 0.01, * *p* < 0.05.

**Table 3:** Analysis for support for radical protests and peaceful protests.

	DV: Support for Radical Actions			DV: Support for Peaceful Protests		
	Model 1	Model 2	Model 3	Model 4	Model 5	Model 6
Gender (F = 0)	0.07 *	0.07 *	0.08 *	0.02	0.03	0.03
Age	−0.01	−0.01	−0.00	−0.01	−0.01	−0.01
SES	−0.14 ***	−0.14 ***	−0.12 ***	−0.16 **	−0.17 **	−0.16 **
Born in HK (No = 0)	0.05	0.06	0.05	−0.02	−0.01	−0.01
Attitudinal support for Anti-ELAB	0.63 ***	0.59 ***	0.53 ***	0.18 **	0.19 **	0.20 **
Participation in Anti-ELAB	0.25 ***	0.22 ***	0.21 ***	−0.04	−0.03	−0.04
Online information exposure	0.04	0.01	0.00	0.38 ***	0.38 ***	0.38 ***
Internet addiction	/	0.09	0.07	/	−0.15	−0.17
FOMO	/	−0.03	0.03	/	0.12	0.11
Depression	/	/	0.13 **	/	/	0.04
Perceived social isolation	/	/	−0.07 *	/	/	0.07
Adjusted R^2^	69.3% ***	69.8% ***	70.9% ***	23.3% ***	23.7% ***	23.7% ***

Note. The entries are standardized coefficients. Missing values are replaced by means. N = 290. *** *p* < 0.001, ** *p* < 0.01, * *p* < 0.05.

**Table 4:** Analysis for Internet addiction, FOMO, depression, and perceived social isolation.

	Internet Addiction	FOMO	Depression	Perceived Social Isolation
Gender (F = 0)	0.06	−0.02	−0.09	0.07
Age	−0.01	−0.03	−0.03	0.00
SES	−0.03	−0.02	−0.14 **	0.05
Born in HK (No = 0)	−0.04	−0.15 **	−0.05	0.01
Attitudinal support for Anti-ELAB	0.30 ***	0.24 ***	0.31 ***	−0.30 ***
Participation in Anti-ELAB	0.26 ***	0.26 ***	0.11	0.07
Online information exposure	0.29 ***	0.29 ***	0.02	−0.02
Internet addiction	/	/	0.28 ***	0.17
FOMO	/	/	0.08	0.15
Adjusted R^2^	46.4% ***	37.6% ***	44.1% ***	5.7% **

Note. The entries are standardized coefficients. Missing values are replaced by means. N = 290. *** *p* < 0.001, ** *p* < 0.01, * *p* < 0.05.
