# Erratum: Tang, G., et al. Politically Motivated Internet Addiction: Relationships among Online Information Exposure, Internet Addiction, FOMO, Psychological Well-being, and Radicalism in Massive Political Turbulence. *Int. J. Environ. Res. Public Health* 2020, *17*, 633

**DOI:** 10.3390/ijerph17082700

**Published:** 2020-04-14

**Authors:** Gary Tang, Eva P. W. Hung, Ho-Kong Christopher Au-Yeung, Samson Yuen

**Affiliations:** 1 Department of Social Science, The Hang Seng University of Hong Kong, Hong Kong, China; garytang@hsu.edu.hk (G.T.); christopherauyeung@hsu.edu.hk (H.-K.C.A.-Y.); 2 Department of Political Science, Lingnan University, Hong Kong, China; samsonyuen@Ln.edu.hk

Due to an error during the editorial process, the article published was not the finalized one [1]. The following details should be corrected.

## 1. Addresses of Some of the Authors

There are some digits after Hong Kong in the authors’ addresses which came from nowhere:

### Published version:

Department of Social Science, The Hang Seng University of Hong Kong, Hong Kong 999077, China

Department of Political Science, Lingnan University, Hong Kong 999077, China

### Corrected version:

Department of Social Science, The Hang Seng University of Hong Kong, Hong Kong, China

Department of Political Science, Lingnan University, Hong Kong, China

## 2. Error in Formatting

The editorial wrongly formatted a paragraph in page 4 and 5 of the published version:

### Published version:

*H2*:  and *H3* try to echo the relationship between online information exposure, Internet addiction, and psychological well-being that has been discussed in psychology. FOMO is also included as it is associated with Internet addiction [38] and it fits the movement context that is full of uncertainties and unexpected incidents. Psychological well-being includes depression and perceived social isolation as they are commonly observed as possible consequences of Internet addiction [29,31]. In the context of a movement, depression was found to be an emotional symptom during and after a protest [39,40], but its role is rarely discussed in social mobilization. In addition, perceived social isolation is not covered in the discussion about mobilization and yet, addictive Internet use can lead to a perception of whether some people are part of the majority or minority in a highly polarized atmosphere during a massive protest. The operationalization of the measurements was adjusted due to the context of massive protest in Hong Kong. This will be explained in the next section.

### Corrected version:

H2 and H3 try to echo the relationship between online information exposure, Internet addiction, and psychological well-being that has been discussed in psychology. FOMO is also included as it is associated with Internet addiction [38] and it fits the movement context that is full of uncertainties and unexpected incidents. Psychological well-being includes depression and perceived social isolation as they are commonly observed as possible consequences of Internet addiction [29,31]. In the context of a movement, depression was found to be an emotional symptom during and after a protest [39,40], but its role is rarely discussed in social mobilization. In addition, perceived social isolation is not covered in the discussion about mobilization and yet, addictive Internet use can lead to a perception of whether some people are part of the majority or minority in a highly polarized atmosphere during a massive protest. The operationalization of the measurements was adjusted due to the context of massive protest in Hong Kong. This will be explained in the next section.

### Published version:

RQ1: RQ1: What is the relationship between depression and support for radical actions?

### Corrected version:

RQ1: What is the relationship between depression and support for radical actions?

## 3. Error in Decimal Places in Table 2 

### Published version:

**Table 2:** Correlations of key variables.

	**(1)**	**(2)**	**(3)**	**(4)**	**(5)**	**(6)**	**(7)**
1. Attitudinal support for Anti-ELAB	--	--	--	--	--	--	--
2. Participation in Anti-ELAB	0.55 ***	--	--	--	--	--	--
3. Online information exposure	0.51 ***	0.40 ***	--	--	--	--	--
4. Internet addiction	0.59 ***	0.54 ***	0.54 ***	--	--	--	--
5. FOMO	0.52 ***	0.49 ***	0.50 ***	0.75 ***	--	--	--
6. Depression	0.57 ***	0.46 ***	0.41 ***	0.58 ***	0.50 ***	--	--
7. Perceived social isolation	−0.10	0.068	0.021	0.13 *	0.15 *	0.01	--
8. Support for radical actions	0.79 ***	0.63 ***	0.47 ***	0.60 ***	0.51 ***	0.60 ***	−0.09

Note. Missing values are replaced by means. N = 290. *** *p* < 0.001, ** *p* < 0.01, * *p* < 0.05.

### Corrected version:

**Table 2:** Correlations of key variables.

	**(1)**	**(2)**	**(3)**	**(4)**	**(5)**	**(6)**	**(7)**
1. Attitudinal support for Anti-ELAB	--	--	--	--	--	--	--
2. Participation in Anti-ELAB	0.55 ***	--	--	--	--	--	--
3. Online information exposure	0.51 ***	0.40 ***	--	--	--	--	--
4. Internet addiction	0.59 ***	0.54 ***	0.54 ***	--	--	--	--
5. FOMO	0.52 ***	0.49 ***	0.50 ***	0.75 ***	--	--	--
6. Depression	0.57 ***	0.46 ***	0.41 ***	0.58 ***	0.50 ***	--	--
7. Perceived social isolation	−0.10	0.07	0.02	0.13 *	0.15 *	0.01	--
8. Support for radical actions	0.79 ***	0.63 ***	0.47 ***	0.60 ***	0.51 ***	0.60 ***	−0.09

Note. Missing values are replaced by means. N = 290. *** *p* < 0.001, ** *p* < 0.01, * *p* < 0.05.

## 4. The Low Resolution of Figure 1

The resolution of Figure 1 in the published version was unclear, and the note was not elaborated.

### Published version:


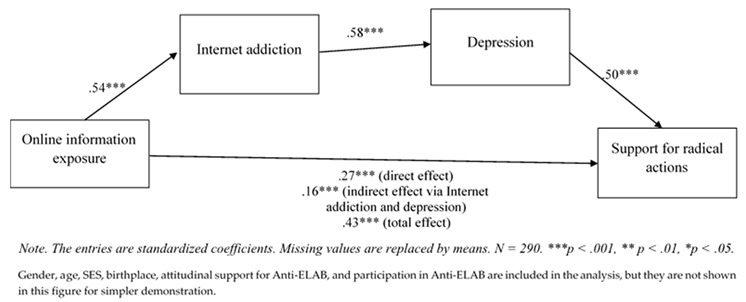


### Corrected version:

**Figure 1:**
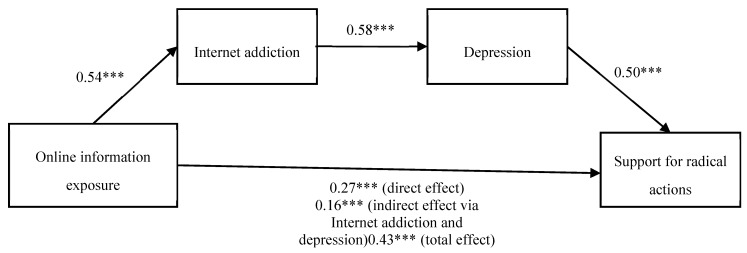
Analysis of the mediating effect of Internet addition and depression on online information exposure and support for radical actions. The entries are standardized coefficients. Missing values are replaced by means. N = 290. *** *p* < 0.001. Gender, age, SES, birthplace, attitudinal support for Anti-ELAB, and participation in Anti-ELAB are included in the analysis, but they are not shown in this figure for simpler demonstration.

## 5. Other Typos 

In the fifth line of the abstract:

### Published version:

The findings reveal the mediating effect of Internet addiction and depression as the main relationship.

### Corrected version:

The findings reveal the mediating effect of Internet addiction and depression on the main relationship.

In the last paragraph in page 6, the fourth line:

### Published version:

It was initially mobilized to urge the government to withdraw the Extradition Bill that could seriously harm the rule of law and liberty in Hong Kong.

### Corrected version:

It was initially mobilized to urge the government to withdraw the proposed amendment of the Extradition Bill that could seriously harm the rule of law and liberty in Hong Kong.

In the second line of “Result” in page 7:

### Published version:

… Anti-ELAB are positively correlated with online information exposure…

### Corrected version:

… the Anti-ELAB movement are positively correlated with online information exposure…

In the last paragraph in page 10, the first line:

### Published version:

Third, being a cross-sectional survey means that the inter-relationships of some variables are… 

### Corrected version:

Third, being a cross-sectional survey, the inter-relationships of some variables are… 

The title of Table 3:

### Published version:

Table 3. Analysis for support for radical protests and peaceful protests.

### Corrected version:

Table 3. Regression analysis for support for radical protests and peaceful protests.

The title of Table 4:

### Published version:

Table 4. Analysis for Internet addiction, FOMO, depression, and perceived social isolation.

### Corrected version:

Table 4. Regression analysis for Internet addiction, FOMO, depression, and perceived social isolation.

These changes do not influence the results, discussion, or conclusions.
